# The role of Nasoalveolar molding: A 3D Prospective analysis

**DOI:** 10.1038/s41598-017-10435-6

**Published:** 2017-08-29

**Authors:** Pang-Yun Chou, Rami R. Hallac, Tochi Ajiwe, Xian-Jin Xie, Yu-Fang Liao, Alex A. Kane, Yong Jong Park

**Affiliations:** 1 Analytical Imaging and Modeling Center, Children’s Health, Dallas, Texas United States; 2 Department of Plastic and Reconstructive Surgery, Chang Gung Memorial Hospital, Taoyuan, Taiwan; 3 Department of Plastic Surgery, UT Southwestern, Dallas, TX United States; 4 Department of Clinical Sciences & Simmons Comprehensive Cancer Center, UT Southwestern, Dallas, Texas United States; 5 Department of Craniofacial Orthodontics, Chang Gung Memorial Hospital, Taoyuan, Taiwan; 6 Department of Orthodontics, Children’s Health Children’s Medical Center, Dallas, Texas United States

## Abstract

Nasoalveolar molding (NAM) is commonly employed to reduce the alveolar segments into proper alignment and to improve nasal symmetry in patients with cleft lip and palate. This study examines the periodical progression of NAM treatment over time. 20 patients with complete unilateral cleft lip and palate were prospectively recruited. A 2 stage NAM treatment protocol was applied. Stage 1 involved adjustment of the alveolar segments (mean age 15.6 days), while Stage 2 added nasal stents and started average 43 days after stage 1. 3D images (n = 241) were obtained prior to NAM initiation and weekly until the end of treatment. The cleft lip area, bilateral nostril areas, and the nostril height and width were measured. Treatment was assessed in the Cleft (C) side and the Non-cleft (N). There was significant difference in the C/N ratio of the nostril area, width, and height at pre-treatment (0.9 ± 0.3, 4.1 ± 1.1, and 0.5 ± 0.2), at the end of stage 1 (1.1 ± 0.3, 2.2 ± 0.6, and 0.8 ± 0.2), and at the end of stage 2 treatment (1.8 ± 0.3, 1.8 ± 0.4, and 1.2 ± 0.1); p < 0.05. Comparative 3D analysis with dense sampling offers a precise methodology for showing effects of NAM treatment. The morphological changes achieved with NAM therapy occur in early treatment phase.

## Introduction

Cleft lip and palate occurs in 1 in 700 US births^1^, making it one of the most common congenital problems. Patients with cleft lip and palate undergo multiple procedures in infancy and adolescence. One major goal of surgical intervention is to restore the aesthetic appearance of the lip and nose by improving the lip scar, nasal tip projection, and symmetry of the nasolabial complex.

Preoperative narrowing of the lip and alveolar segments helps to reduce tissue tension and is thought to improve surgical outcome by minimizing wound healing disturbances and scarring^2^. In 1999, Grayson proposed a pre-surgical nasoalveolar molding (NAM) procedure that consists of infant premaxillary orthopedics and nasal molding to reduce the severity of the cleft lip deformity and improve the symmetry of the nose prior to surgical repair^3–5^. Some clinicians believe NAM is an indispensable technique to reduce the severity of the initial defect, aligning alveolar segments, normalizing lower lateral nasal cartilage position, and expanding nasal lining^6, 7^. Despite noting a paucity of high-level evidence, a recent systematic review found that protocols involving NAM may improve nasal symmetry in the first 1 to 6 years^8^. Several studies have evaluated the outcomes of NAM using 2D linear measurements^9–13^, and others have used landmark-based 3D analysis to describe outcome^2, 14, 15^. However, we can find very few studies addressing the progressive temporal morphological changes in the lip and nose which occur with NAM treatment.

3D photography (stereophotogrammetry) is a nonionizing, noninvasive, and fast image capture (about 1.5 ms per frame) technique^16^ that has recently gained interest in pediatric craniofacial imaging^17–21^. Accurate 3D surface image measurements are important factors for the analysis of deformities and surgical planning. Stereophotogrammetry has shown to be reliable and accurate when compared to manual anthropometry^16, 22, 23^. The aim of our study is to assess the 3D progressive changes of the lip and nose during NAM in patients with complete unilateral cleft lip and palate.

## Results

Image analysis was performed on 20 patients with unilateral complete cleft lip and palate who underwent NAM treatment. The mean age of the study population at inception of NAM treatment was 15.6 days (median 14 days) (range 5 to 57 days) with male: female distribution of 14:6. Twelve patients had a left-sided cleft and eight had a right-sided cleft. The racial distribution of the population was 16 Hispanic, two Caucasian and two African-American.

Subjects were imaged weekly for about 10 weeks (range 8 to 16 weeks) and all acquired images (a total of 241 images) were of high quality. First stage NAM (alveolar molding alone) ranged from 21 to 84 days with a mean of 43 days. Second stage ranged from 28 to 119 days with a mean of 55 days.

**Figure 1:**
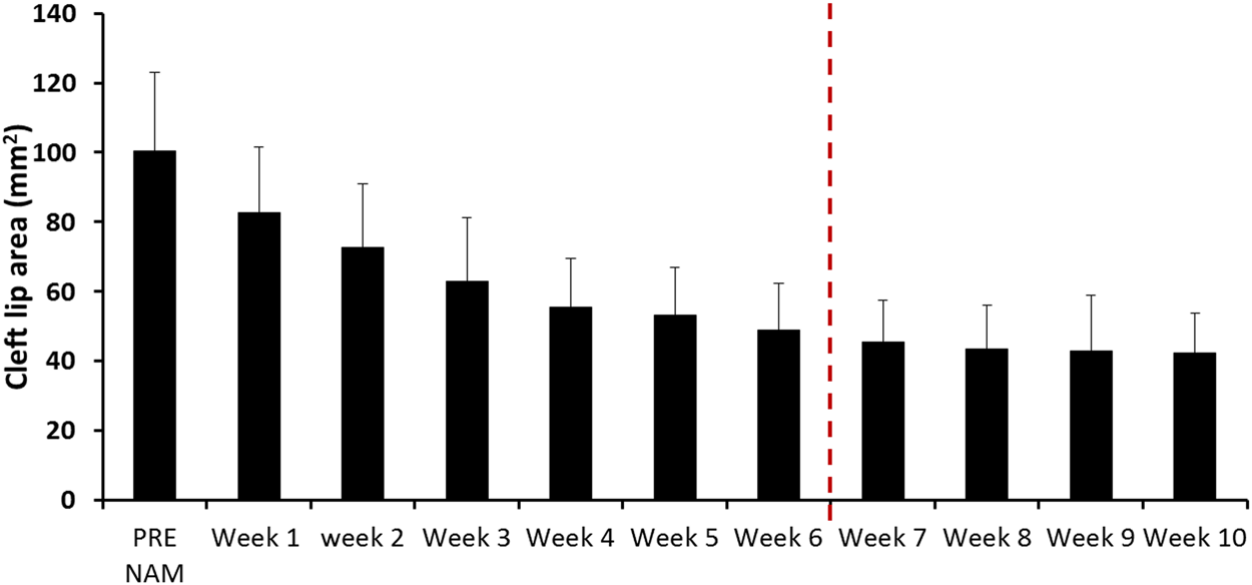
Lip diastasis area over time, with weekly sampling. Dotted vertical line shows junction of first and second stage NAM therapy. Note rapid pace of changes in early stage 1 therapy, with nearly half of the total lip area decrease occurring within 2 weeks of treatment onset.

The mean cleft lip area was 100.4 ± 22.7 mm^2^ before treatment, which decreased to 48.8 ± 13.4 mm^2^ after the completion of stage 1 and 42.4 ± 11.3 mm^2^ at the end of treatment (10^th^ week). Typically, narrowing of the cleft diastasis occurred rapidly, with approximately half of the total amount of improvement occurring in the first 2 weeks of treatment. The remaining half of the improvement was more gradual, spread evenly over the remaining 8 weeks of treatment (Fig. 1).

### Overall comparisons

**Figure 2:**
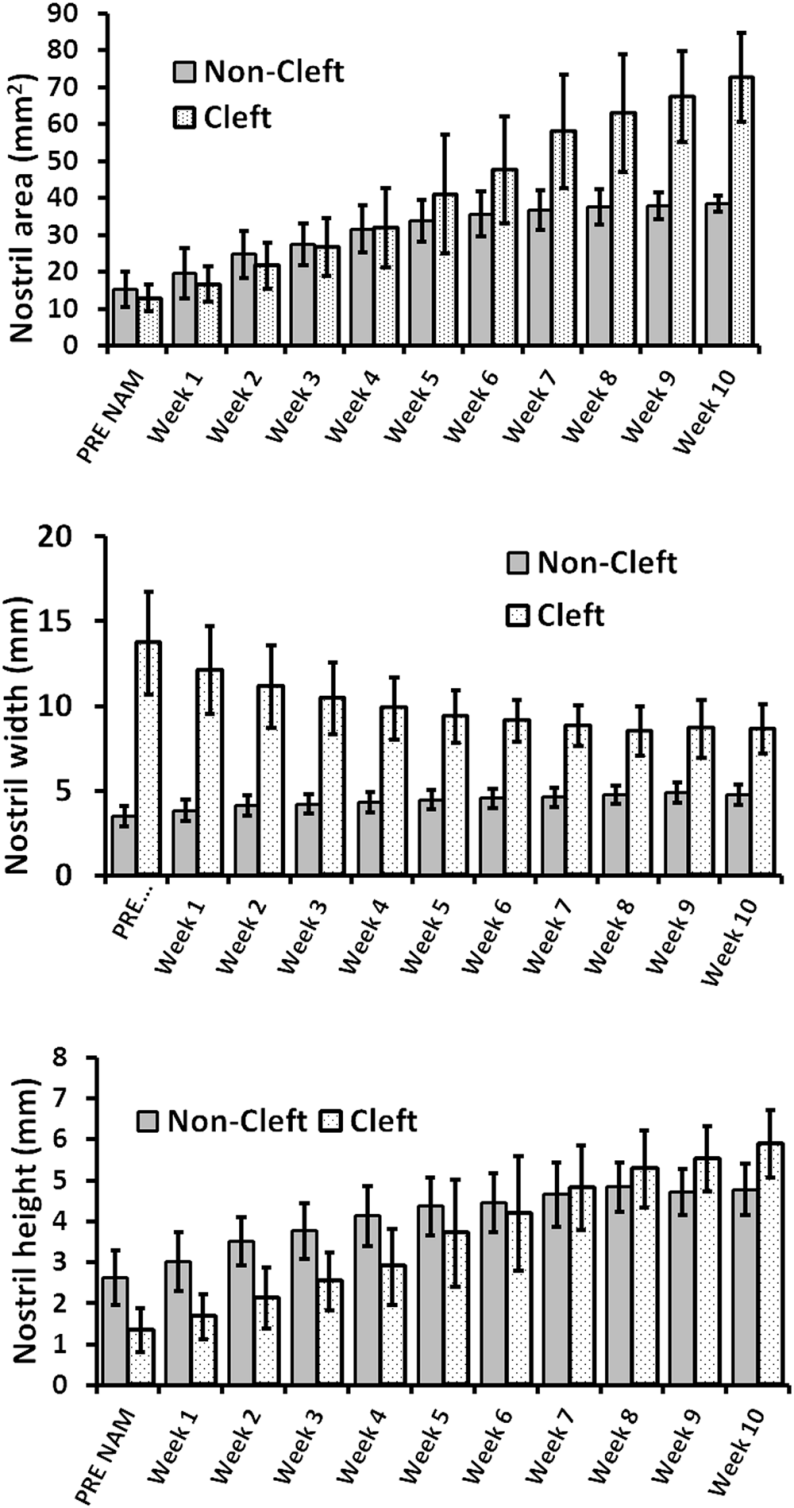
Breakdown of progressive changes in nostril area, height, and width over time. Nostril area and height on both the cleft and normal sides increases, while the cleft nostril width decreases with NAM treatment. Normal side nostril width gradually increases.

On average, the cleft nostril area increased in direct proportion to duration of NAM treatment. During the first stage of NAM, the cleft nostril area increased at the same rate as the non-cleft side nostril but during the second stage it increased significantly faster than the non-cleft side. The non-cleft side nostril area increased only during the 1^st^ NAM stage. The cleft nostril height increased over the entire duration of NAM therapy, while the non-cleft nostril height increased predominantly in stage 1. The major decrease of the cleft nostril width occurred in stage 1, while the non-cleft nostril width increased slowly over the full span of treatment (Fig. 2).

**Figure 3:**
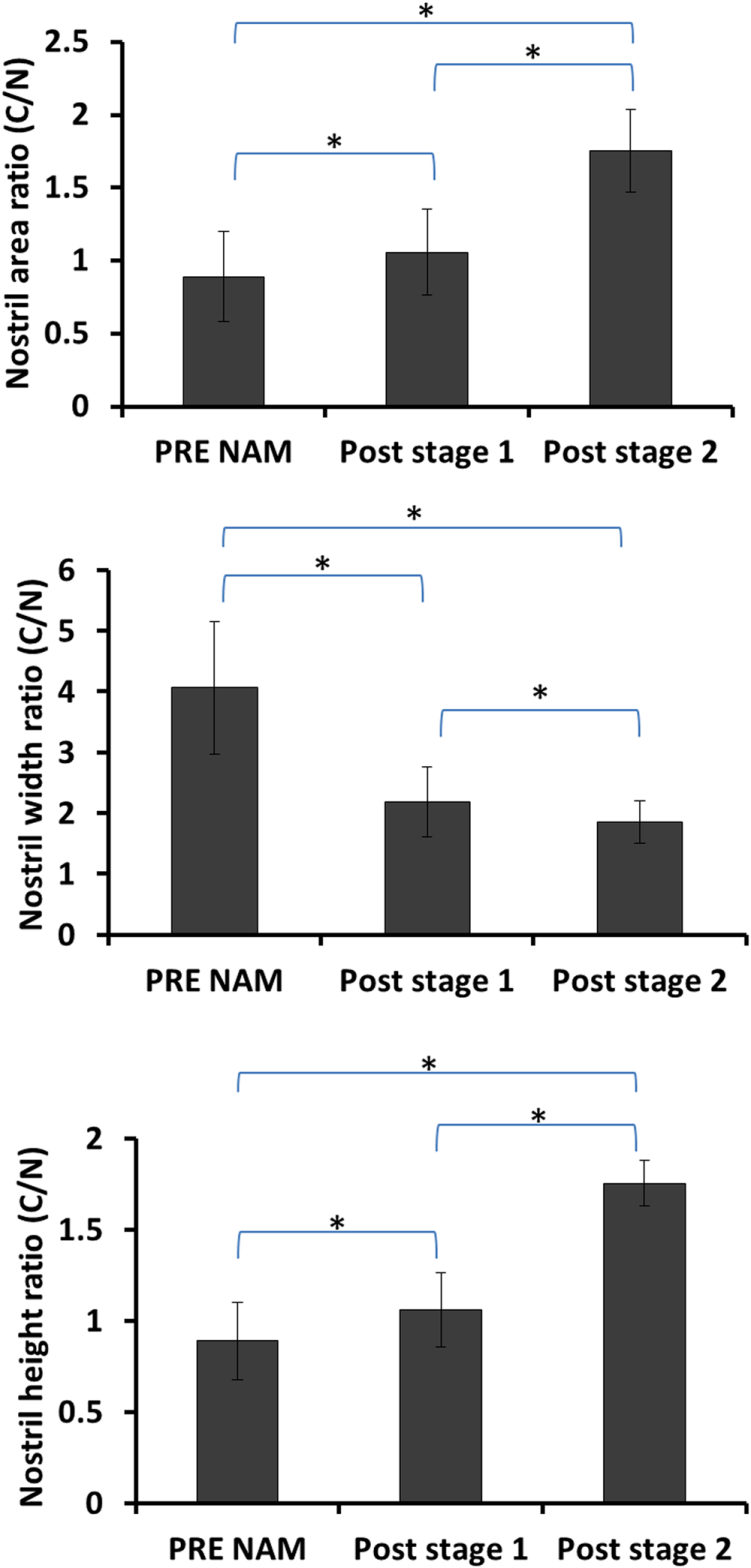
Statistical comparison of nasal outcomes by stage of treatment measures. Significant differences (p < 0.05) indicated by *.

The differences in outcomes by stage of treatment were tested for significance by calculating ratios of cleft to non-cleft values (C/N) for nostril area, height, and width (Fig. 3). These ratios were found to be statistically different between each stage of treatment for all outcome measures. The C/N ratio of nostril area and nostril height improved more in the second stage of therapy, while nostril width increased more in the first stage than in the second.

### Group comparisons

**Figure 4:**
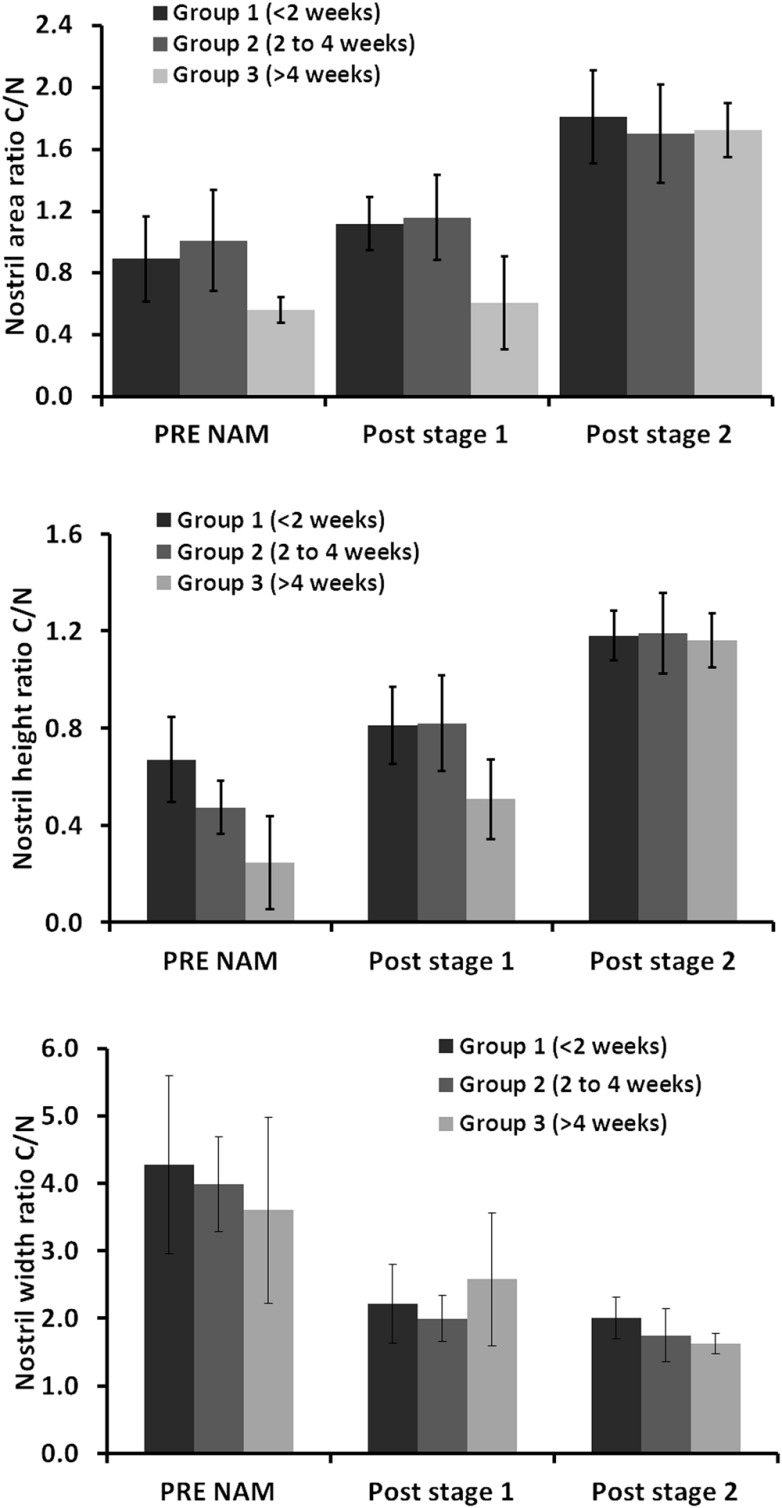
Effect of age at onset of treatment.

There were 9 patients in group 1, 8 patients in group 2 and 3 patients in group 3. The C/N ratios of nostril area at post stage 2 are 1.8 ± 0.3, 1.7 ± 0.3, and 1.7 ± 0.2 in group 1, 2, and 3. The C/N ratios of nostril height at post stage 2 are 1.2 ± 0.1, 1.2 ± 0.2, and 1.2 ± 0.1 in group 1, 2, and 3. The C/N ratios of nostril width at post stage 2 are 2.0 ± 0.3, 1.8 ± 0.4, and 1.6 ± 0.2 in group 1, 2, and 3 (Fig. 4). Owing to the little case number in group 3, no statistics are performed for the comparisons of the three groups in different stages. Even though, the C/N ratios of the three groups are much different in the pre-NAM treatment and post-stage 1, the ratios are similar at the end of the NAM treatment, indicating equivalent outcome.

## Discussion

3D imaging techniques have been widely adopted by the craniofacial community and have been shown to be accurate when compared to manual anthropometry^16, 22, 23^. However, relatively fewer 3D studies related to NAM have been published^6, 15, 24^ and 2D methods are more numerous^9–13, 25^. To our knowledge, this is the first study to prospectively and rigorously analyze the 3-dimensional progressive changes with dense acquisition of periodic imaging data in children with unilateral complete cleft lip and palate who underwent presurgical NAM treatment prior to primary lip repair.

The overarching goal of this study was to understand how rapidly NAM produces changes. During the first stage of treatment, nearly 50% reduction in the diastasis of the lip was observed in the first two weeks. Stability was reached at 6 to 7 weeks of treatment when the second NAM appliance was delivered, as the treatment focus shifts to molding nasal cartilage and lengthening of the columella.

The rapid decrease in the cleft nostril width occurs concomitantly with approximation of the alveolar segments during the first stage of NAM treatment. With alveolar segments alignment, the distance between alar bases and the diastasis of the lip are reduced. Yet it was intriguing to observe that the nostril width asymmetry between cleft and non-cleft sides continued to decrease in the second stage of NAM treatment. It seems that this ongoing decrease in cleft nostril width during the second phase results from reshaping of the nasal cartilages towards normal. Similarly, there was significant correction of nostril height during the first stage, although correction of nostril height is considered a primary treatment goal of the second stage, when nasal stents are employed as a feature of the appliance. These results indicate that alveolar segment molding not only reduces the distance between segments, but also elevate the immature and flat lower lateral cartilage on the cleft side.

Significant changes in nostril area occurred in the first and second stages of NAM treatment. In the first stage, non-cleft side nostril area increases as the alveolar segments are approximated and lip diastasis decreases. In addition, the cleft nostril area increases as the cleft nostril height increases despite the decrease in cleft width which occurs in this phase. In the second stage, non-cleft nostril area remains stable but cleft side nostril area increases. This occurs as the cleft nostril height increases while the width remains nearly unchanged.

Noting that significant changes occur at early stage of NAM treatment prompts the question of the advantage of initiating the NAM treatment at young age. In this preliminary study, we noticed that children older than a month of age at initiation of NAM had more asymmetry with respect to nostril area after stage one, which could be attributed to the relatively shorter stage one treatment for this age group. Overall, there were no significant differences in cleft to non-cleft ratios at completion of therapy. Matsuo reported that delay in NAM treatment might influence outcome due to decreasing plasticity in the nasal cartilage as the infants age^26^. Shetty concluded most of the benefit of NAM is obtained when NAM is applied before 1 month of age^12^. In our study the three patients over a month in age at time of commencing therapy eventually obtained equally good outcome as the other children who had earlier initiation. Again, further study is warranted due to small sample size of this age grouping. Despite the good outcome in these patients, we concur that earlier treatment is prudent^27^. We believe that earlier treatment of congenitally deformed cartilage structures favors earlier molding intervention, as has been established in neonatal ear cartilage molding^28, 29^.

For nasoalveolar molding therapy to succeed (and for complete data collection in this study), parent and caretaker compliance with frequent visits is crucial^30, 31^. Chen *et al*. suggested that reduced frequency of adjustments results in decreased cost of care^11^. In our study, we did not test adjustment frequency but the methodology used here would be applicable in a well-designed prospective study.

In contrast to our progressive analysis with dense sampling, most of the literature compares only the pre- and post-NAM morphologies^9, 10, 12, 27^. Our study demonstrates the tempo and degree of progressive changes in nasal symmetry, and was able to document complete nasal correction or even overcorrection prior to primary lip repair. According to van der Heijden *et al*., the evidence that NAM achieves nasal symmetry is not compelling^32^. On the other hand, Liou *et al*. suggests overcorrection to compensate relapse and differential growth after primary lip repair^33^. While this study does not address the postsurgical value of preoperative NAM, the generally agreed upon presurgical goals of NAM treatment (i.e. reduction in the size of alveolar gap and improvement in nasal symmetry) were achieved in this study^34^. Long-term follow up of the cleft patients receiving the NAM treatment prior to lip repair should be observed to ensure the durability of the improvements noted with NAM treatment. Further studies might be focused on enrolling a control group to tease apart the effects of growth vs. NAM treatment, and assurance that adequate statistical power is achieved.

Serial 3D analysis based on dense samplings provides a precise methodology for evaluation of NAM treatment. Early achievement on the morphologic change with NAM therapy could be expected. NAM achieved significant improvement in measures of cleft width and nasal form in all patients, regardless of the initial age of treatment.

## Materials and Methods

Institutional Review Board (IRB) approval was obtained by UT Southwestern medical center and all methods were performed in accordance with the relevant guidelines and regulations. In addition, informed consent was obtained for both study participation, and publication of identifying images. 20 patients with unilateral complete cleft lip and palate without other facial deformity were recruited to the study. The children’s ages ranged from 1 to 8 weeks old.

### Presurgical Nasoalveolar Molding

A two-stage modified NAM protocol was performed. The patients visited the Craniofacial Team Care Clinic weekly for NAM adjustments.

Preparations for NAM appliance was made during the first appointment, which consisted of (1) parental intake interview, (2) intraoral and extraoral 3D stereophotogrammetric imaging, and (3) intraoral impression with putty (PVS, polyvinylsiloxane) impression material. These impressions were made by the orthodontist in the outpatient clinic without the use of anesthesia. The NAM appliance was applied at the second appointment (7 days post impression). Parents were instructed to leave the NAM appliance in place at all times except for cleaning.

The NAM procedure in our study consisted of two stages. Stage 1 starts with alveolar molding alone, using a first appliance without a nasal stent and with dental floss threaded to the appliance for safety to prevent aspiration. The appliances were adjusted weekly to mold the alveolar segments by selective grinding and application of soft acrylic. Stage 1 ends when the gap between the cleft alveolar segments became smaller than 5 mm (~6 weeks) or lip segments could be approximated without much tension. Stage 2 began with the use of a second appliance, providing nasal stents to correct the nose projection and asymmetry. The appliance was retained with denture adhesive and the lip is taped with 3 M micropore tape at all times. A small amount of Mastisol was applied to the lip to increase tape retention.

**Figure 5:**
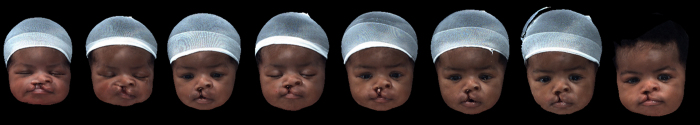
Sample progression of serial 3D images and associated changes observed during NAM therapy for UCLP.

The patients underwent 3D stereophotogrammetric imaging (3dMD, Georgia, USA) weekly (without NAM appliances in place) until the end of treatment (Fig. 5). A total of 241 images were obtained for all patients. Every effort was made to acquire the images with a neutral facial expression.

### Treatment History

Each patient’s clinical notes were examined and details of nasoalveolar molding treatment were recorded, including age at beginning of treatment, presence of complications (e.g., facial irritation or oral mucosal ulceration), and treatment duration.

### 3D image analysis

The images were converted to stereolithography file format (.stl) using 3dMDVultus software (3dMD, Georgia, USA) to prepare them for analysis. Cleft lip area, nostril area, nostril height and nostril width were measured using 3-Matic software (Materialise, Belgium).

**Figure 6:**
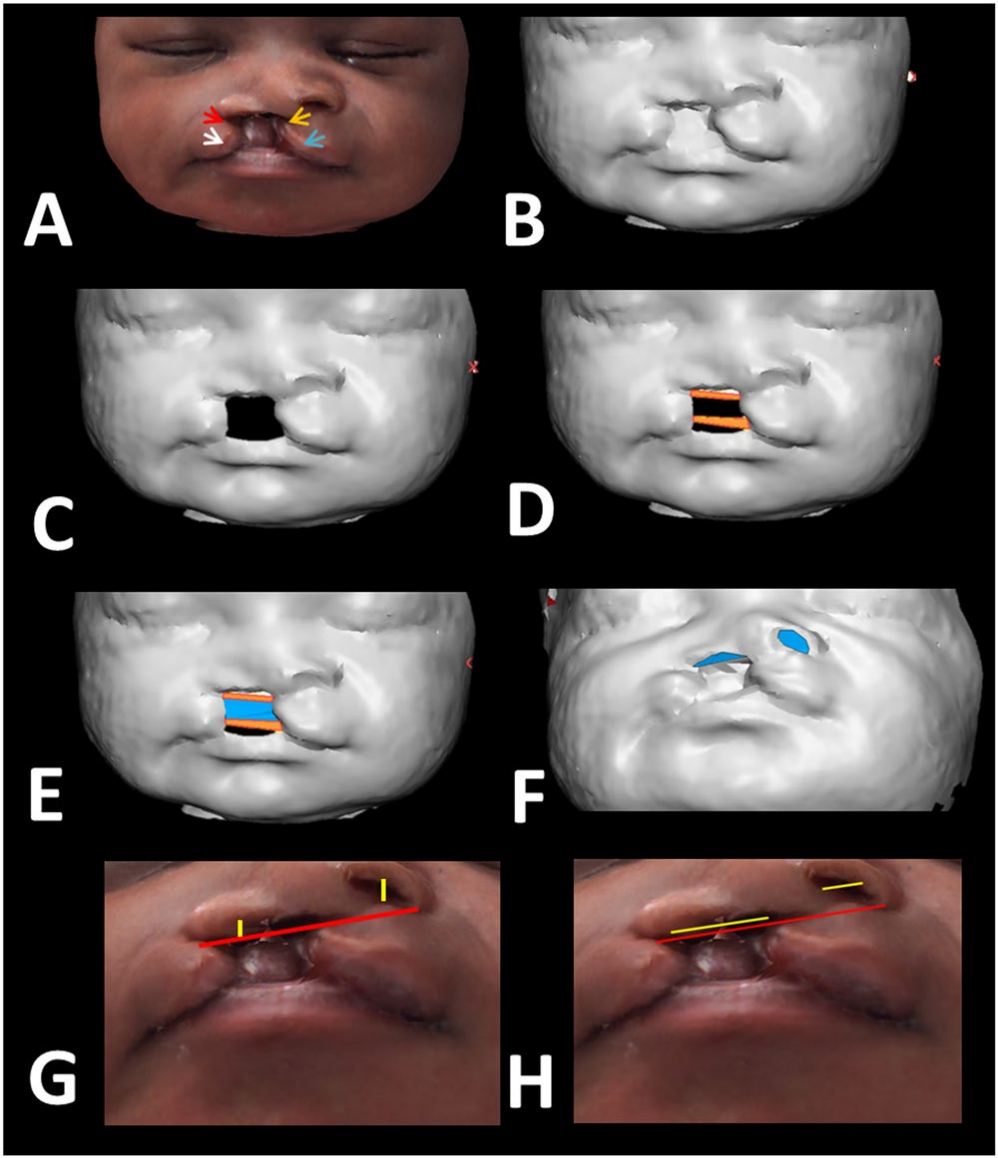
Lip diastasis and nostril measurements performed: (**A**) stereophotogrammetric image with texture map showing the four major landmarks, white = labiale superius (ls), blue = crista philtri landmark (cph), yellow = subnasale’ (sn’) and red = subalare (sbal). (**B**) Stereolithography converted image (**C**) defect cropped using four landmarks. (**D**) Upper and lower boundary limits were defined by bridging sn’ and sbal, ls and cph. (**E**) The cleft area was filled (blue) using the boundaries defined by D. (**F**) Nostril areas defined in blue. Measurement of nostril (**G**) height and (**H**) width in 3dMDvultus.

To calculate the area of the cleft diastasis, a contour that surrounds the lip defect region was determined by recognizable landmarks, including: labiale superius (ls), crista philtri landmark (cph), subnasale’ (sn’) and subalare (sbal)^35^. The gap was filled with a mesh surface and its area was calculated. Nostril area, width, and height were also calculated. The method utilized involved creation of a reference line defined by spanning the alar bases, which also intersected the base of the columella as closely as possible. The cleft nostril area was then measured by closing the cleft nostril above the reference line as it intersected with the arc of the cleft side ala. The non-cleft nostril area was measured by simply inscribing the closed oval defined by its border. Nostril width was determined as the maximal width of the nostril at a parallel line segment above the reference line, while nostril height was measured by the length of a vector from the highest point of the nostril dome and the reference line (Fig. 6). These measurements were obtained weekly throughout NAM therapy.

Patients were divided into three groups according to the age at initiation of NAM treatment: Group 1 was less than 2 weeks of age, Group 2 was 2 to 4 weeks of age, and Group 3 was older than 4 weeks. NAM treatment progression was assessed at three critical intervals: pre-NAM treatment, post stage 1, and post stage 2.

### Statistical Analysis

Paired *t*-tests were performed to compare NAM progression at different stages and treatment age initiation. Reliability testing was performed on 5 patient datasets (50 images). A single observer obtained measurements on separate days with a three-month interval between landmarking sessions. Intraclass correlation coefficient (ICC) indicated a high reliability ICC = 0.9991 with 95% CI from 0.9989 to 0.9992 and a p-value < 0.0001.

Kolmogorov-Smirnov analysis was performed to test our data for normality. The results indicated that we may assume the data are normal (either pre, post 1 or post 2). Statistical analyses were performed using IBM SPSS for Windows, Version 19.0 (IBM Corp., Armonk, N.Y.). The significance level (*α)* was set at 0.05 (5%).
